# Infantile Eclampsia1Read before the Buffalo Academy of Medicine, January 13, 1903.

**Published:** 1903-05

**Authors:** A. J. Colton

**Affiliations:** Buffalo, N. Y., Pediatrist to Buffalo Hospital, Sisters of Charity, 25 East Ferry Street.


### Infantile Eclampsia.[1]

By A. J. COLTON, M. D., Buffalo, N. Y.,
Pediatrist to Buffalo Hospital, Sisters of Charity.

ECLAMPSIA is a term often used synonymously with convulsion. It may be defined as a violent, irregular, paroxysmal contraction of certain muscles,—either tonic or clonic in character, though mostly clonic and accompanied by partial or complete loss of consciousness. The movements may be general or confined to one side of the body; may involve a group of muscles or a large number; or, one form may merge into another. The cause of the convulsions may vary from the most trivial reflex disturbances to a very serious and profound cerebral lesion. In this paper, I shall refer more particularly to those forms of eclampsia in which there is no apparent lesion of the nervous system. I shall, therefore, not consider it as a disease, *per se*, but merely as a symptom of a great variety of morbid conditions, affecting the most diverse portions of the animal economy. But while eclampsia is only a symptom, it is one of so pronounced a character that it merits and, indeed, requires special consideration regarding its points of origin, etiology, nature and treatment.

In the infant the motor neuroses predominate largely over the sensory; hence neuralgia and disturbances of sensation are rare, while all forms of convulsive disorders are exceedingly frequent, especially during the first three years of life. A large proportion of the convulsive disorders of infants, whether local or general, are reflex in their origin, being due to some eccentric irritant acting upon the peculiar impressionable nervous system of the infant. Eclampsia in infants depends upon abnormal conditions of the nervous system, concerning which we know very little. The causes of these abnormal conditions are legion. It may be caused by reflex irritation of whatever nature, proceeding from the central or peripheral nervous system; abnormal conditions of the blood, as found in acute specific and other fevers; in diseases of the kidneys and acute poisoning; autoinfection; ptomaines; by an anemic state of the brain, such as follows severe hemorrhages and profuse diarrhea; by the interference with proper oxygenation and decarbonisation of the blood, as in asphyxia, or by any profound interference with the circulatory or respiratory system.

1. Read before the Buffalo Academy of Medicine, January 13, 1903.

Acute intracranial pressure may cause convulsions by producing a capillary anemia, also, acute cerebral hyperemia may act in a similar manner. Sudden high bodily temperature in itself is a frequent source of convulsions, which I have several times witnessed.

Worms and dentition were once thought to be the most frequent source, and it is probably true that they are occasionally a cause, but there is no doubt that this source has been greatly overdrawn by our forefathers in medicine, as well as the mothers of the present day.

Children in good health cut teeth without the slightest nervous disturbances, and how many of us know of children passing worms without the slightest symptom and observed by the mother only by chance. And, again, what physician has not seen an ill-nourished, rachitic infant, become feverish, cross and irritable; in fact, on the verge of a convulsion and possibly in one, from a tender and swollen gum, and become almost immediately relieved by lancing. This I believe, though, to be an unusual source of eclampsia, and occurs only in those who are rachitic and ill-nourished. Instances of phymosis and adherent prepuce have been known to act as a cause. Age must certainly be considered as an important factor in the etiology of convulsions, it being a well-known fact that children under two years are more frequently attacked than those beyond that age.

Sex is said to have a causing influence, females being more prone to convulsions than males. Heredity may have its effects. A child cannot inherit convulsions but it may inherit such a condition of the nervous system as to predispose to them. Rachitis is generally believed to be a frequent predisposing factor in causing convulsions. Laryngismus, stridulus, tetany, and general convulsions are the common types of eclampsia in this disease. Typhoid and intermittent fevers, pneumonia, whooping cough, toxemia from any cause; malformation and diseases of the heart; irritation of the peripheral nerves, such as burns, stings, bites; also earache, independent of any meningeal trouble, are among causes. Fright and other strong emotions are said to be sources, though I have never observed them.

The various causes heretofore given have been largely of a reflex type, but we may and do have convulsions caused from some gross lesion of the brain or spinal cord, such as the various forms of meningitis, cerebral hemorrhages, emboli and thrombosis, anterior poliomyelitis, and the various acute spinal affections, aside from those causes produced by direct injuries to brain and spinal cord. In my experience, by far the most frequent and fertile source of all infantile convulsions is due to some irritating disturbances along the alimentary tract, such as that produced by over and improper feeding. The convulsion may be caused directly from the irritating effect of the indigestible food, or it may be caused indirectly by setting up fermentation and decomposition, causing an intoxication or autoinfection, gastritis, gastroenteritis, enterocolitis, dysentery, diarrhea, cholera infantum and the like, and in fact, most of the various affections of the alimentary tract.

That improper feeding is a frequent cause may be shown by citing a few interesting and typical cases.

Case I.—Baby S., aged 18 months; called to see in great haste and found in a terrific clonic convulsion, involving most of the muscles of the upper and lower extremities and face. Knowing the mother and her mode of caring for and feeding her children, I proceeded at once to empty the intestinal canal by irrigation, which done, brought forth undigested sauerkraut, wieners and other indigestible food products that would have put to blush a free lunch counter. Immediately on its removal the convulsion ceased.

Case II.—Baby R., about 2 years; summoned in great haste and found baby in a general convulsion. I at once proceeded to empty intestinal canal by irrigation and brought forth a quantity of undigested beans and the like. The convulsion immediately ceased on its removal.

Case III.—Baby M., about 2 years; summoned in great haste, but not being at home, another nearby physician was called. I, however, arrived before the child was out of its convulsion and before the other physician had instituted treatment, but just as he was about to give 1-16 gr. morph. subcutaneously. Knowing the patient and parents, I suggested that we try to get at the source of the trouble and recommended irrigation of the bowels, to which he consented, and was immediately done, when, behold, a voluminous discharge of very offensive undigested foods of all kinds and description issued forth. The convulsion at once ceased.

Case IV.—Baby F., about 3½ years; called to see and found in a violent convulsion. I at once began to irrigate bowels and emptied them thoroughly, but child did not come out of the convulsion. I then had cold applications applied to head, hot mustard bath to extremities, and injected about 10 gr. each of triple bromide and chloral per rectum. The convulsion ceased in about half an hour afterward.

In the first two cases convulsions undoubtedly were produced by direct irritation of the indigestible foods; the third was probably due to toxemia set up by the decomposed and fermented foods; the fourth proved to be due to a tubercular meningitis. The pathology I will leave with my hearers to read for themselves.

*Symptoms of an oncoming convulsion.*—The child sleeps with its eyes half open, showing the white sclera beneath the upturned cornea, becomes restless, starts and cries out in its sleep, grits its teeth, twitches its mouth and occasionally a smile (*risis sardonicus*) passes across its face. There may also be a tendency to flexion of the thumbs in the palms. It gazes into vacancy, usually to one side and upward with an expressionless face. The pupils change frequently and are usually widely dilated.

*Treatment.*—When a physician is called in to see an infant in convulsions, he has no time to spare in getting history of the case. The infant is in imminent danger of asphyxia from paralysis or spasm of respiratory muscles or glottis; hence, what he does must be done quickly. As at least a very large percentage of all infantile convulsions is due to improper and overfeeding, I attempt to at once empty the intestinal tract as soon as possible. To wait for a dose of calomel or castor oil to act is out of the question, for the infant could die a score of times before cathartics would have time to act; furthermore, the infant is unable to swallow.

If I find the infant in imminent danger of asphyxia, I administer a small amount of chloroform to relieve spasm, and have cloths rung out of warm mustard water applied to extremities, while an enema of soap suds and water, with a few drops of turpentine, is being prepared. If a fountain syringe is present I use that—first gently stretching the sphincter so that the enema will be easily returned and avoid danger of over-distending the bowel. I then proceed to irrigate until the offender has come away. If the spasm is caused from fermented or undigested foods, it will come out of the convulsion almost as quickly as the offending material is detruded. Should the spasm not cease after thorough evacuation of bowels, I inject into the rectum with a small piston syringe, which I always carry in my medicine case, a solution of triple bromides and chloral, about 10 gr. of each, for an infant of 2 years. In addition to this, the child is put in a warm bath and ice bag to its head, if the infant has a high fever. Should not the convulsion, which you are treating, be caused from offending material in the intestinal canal, you have done no harm by irrigating, for should it be caused from an oncoming fever, producing the convulsion either from high temperature or a toxic state of the blood, you have helped to reduce the fever; caused a depletion of the intestinal capillaries and thereby throwing off more or less toxic material; or, if due to cerebral hyperemia or meningitis, you have done good by producing a revulsive action, drawing the blood from a congested brain, which is the usual condition during convulsions. If you see the infant before it has a convulsion, but in imminent danger of one, emetic doses of ipecac or turperth mineral is admirable treatment in connection with irrigation. If you think the drug will not have time to act before an eclamptic seizure, tickle the fauces with the finger until emesis is produced. If the nerous disturbances and fever are caused from some indigestible food in the stomach, the fever will soon subside and the nervous symptoms disappear, if there has not been produced too great a congestion or toxemia; then nerve sedatives and mild antiseptics will be required.

After the infant is out of the convulsion, then get a history of the case and study the cause. If you are still convinced that it was due to an intestinal toxemia or from indigestible and decomposed food, administer a cathartic, such as a dose of calomel or castor oil, followed by stomachic and nerve sedative, such as salol, and bismuth and triple bromides. All solid foods should be withheld for twenty-four hours, giving pure cold water, such as crystal water, albumin water, rice or barley water, or freshly prepared sterile whey made with pepsin. The infant should be kept in a darkened room and absolutely quiet.

If you find that the eclampsia has been due to a tender and swollen gum, lance it; if to an adherent prepuce, break up adhesions and wash away the smegma; if due to a long and tight foreskin, circumcise; or, if due to rachitis, correct the diet, give bone salts, cod liver oil, massage and strict observance of its hygiene; if worms are found, get rid of them; or, in other words, search for the cause and then endeavor to correct it.

To summarise, when called to see an infant in convulsions, if you are in doubt as to the cause, irrigate the bowels at once, at the same time applying counter irritation to extremities, such as cloths wrung out of hot mustard water and ice to the head; if indicated by a high temperature, a general warm bath may be given at the time of irrigation. Should the convulsion be not due to indigestible or fermented food, no harm has been done by irrigating the bowels, and in many instances a human life will have been saved.

25 EAST FERRY STREET.

